# Empowered yet dehumanized: perceptions of women’s attractiveness in the context of gender earnings inequality

**DOI:** 10.1186/s40359-025-03306-7

**Published:** 2025-08-19

**Authors:** Lijuan Xiao, Lei Cheng, Fang Wang, Junhua Dang

**Affiliations:** 1 Institute of Social Psychology, School of Humanities and Social Sciences, Xi’an Jiaotong University, Xi’an, 710049 China; 2 Beijing Key Laboratory of Applied Experimental Psychology, National Demonstration Center for Experimental Psychology Education (Beijing Normal University), Faculty of Psychology, Beijing Normal University, Beijing, 100875 China; 3 School of Psychology, Fujian Normal University, Fuzhou, 350007 China; 4 Department of Surgical Sciences, Uppsala University, Uppsala, Sweden

**Keywords:** Attractiveness, Empowerment, Dehumanization, Gender earnings inequality

## Abstract

**Background:**

Gender earnings inequality remains a significant issue in the labor market. In response, women may perceive attractiveness as a potential resource. However, the effectiveness of attractiveness may vary depending on whether it is assessed from a first-person or third-person perspective.

**Methods:**

To explore how attractiveness is perceived in the context of gender earnings inequality, we conducted two studies. Study 1 examined women’s self-perceptions of their attractiveness when faced with gender earnings inequality. Study 2 investigated how third-person observers perceive women’s emphasis on attractiveness in the same context.

**Results:**

Study 1 revealed that women reported feeling empowered by their attractiveness. However, Study 2 found that third-party observers perceived women’s emphasis on attractiveness as a form of self-dehumanization. Specifically, participants viewed these women as less human, less empowered, more susceptible to sexual objectification in daily life, and less likely to be chosen as friends in the gender earnings inequality context.

**Conclusion:**

These findings provide evidence for the perceived empowering function of women’s attractiveness as a response to gender earnings inequality while also highlighting a critical discrepancy between women’s self-perceptions and third-party evaluations of attractiveness.

**Supplementary Information:**

The online version contains supplementary material available at 10.1186/s40359-025-03306-7.

## Introduction

Enhancing one’s appearance has become increasingly common. Reports suggest that the demand for plastic surgery has surged in recent years, with the global market projected to reach an estimated value of 2 trillion United States Dollars by 2026 (Zion Market Research 2020). People may internalize cultural beauty ideals and evaluate their own bodies from a third-person perspective, focusing more on their appearance (e.g., sex appeal) than on inner attributes (e.g., competence). These appearance concerns and body surveillance are conceptualized as self-objectification, and women are believed to be more vulnerable to self-objectification than men (Fredrickson and Roberts 1997; Roberts et al. 2018).

The prevalence of human tendencies for appearance enhancement suggests that attractiveness serves essential functions, such as increasing reproductive fitness or social status (Kowal et al. 2022). Postfeminist neoliberal economies, which have encouraged feminine subjects to reconsider their subjectivity, particularly their autonomous agency and empowerment (Gill 2007), have led to the belief that attractiveness can function as a form of capital for women to gain power and rewards (Erchull and Liss 2013, 2014; Gill 2008). However, the discourse of individualism, choice and empowerment appears questionable, as women continue to be constrained by inequality and power imbalances (Gill 2007).

It remains unclear whether women and third-party observers perceive a woman who explicitly leverages her attractiveness to achieve goals in the same way. Specifically, this raises the question of whether women who feel empowered by their attractiveness in gender-disadvantaged situations are perceived as empowered or, paradoxically, as victims of cultural beauty ideals. In this study, we aim to investigate whether women and third-party observers share the same perception of the impact of attractiveness on women.

### Attractiveness buys women both partners and jobs in the gender earnings inequality

Gender earnings inequality refers to the economic disparity between women and men in the workplace, which is typically measured by comparing the average hourly, weekly or annual earnings of women to those of men and examining the earnings gap between genders (Blau and Kahn 2003). Gender earnings inequality remains a significant issue in the labor market, as highlighted by Claudia Goldin, the Nobel Memorial Prize recipient in 2023. In 2024, only 60.5% of the gender gap in economic participation and opportunity had been closed, with an estimated 152 years needed to fully close the gap (World Economic Forum 2024). Furthermore, women are more frequently affected by the gender earnings inequality, especially in the 10 to 16 years following MBA completion (Bertrand et al. 2010).

The gender disparity in earnings inequality may drive women to compete for resources, both economic and social, in both private and public spheres. Among the available strategies, cultivating an attractive appearance is often perceived as one of the most accessible options (Gill 2008). Since social power stems from an individual’s asymmetric control over resources (Fiske and Berdahl 2007), women’s relative economic disadvantage can place them in the inferior status compared men. Consequently, gender earnings inequality may exacerbate women’s economic dependence on their male partners in heterosexual relationships. This aligns with the views of evolutionary psychologists, who argue that women’s attractiveness plays a critical role in mating (Buss 1989). Appearance enhancement can be used as a self-promotion strategy, potentially improving women’s reproductive success (Davis and Arnocky 2022). Similarly, sexual economics theory (Baumeister and Vohs 2004) likens mating to a marketplace, where women are sellers of sex and men are buyers, with an attractive appearance commanding a higher price for women. Empirical evidence also underscores the importance of attractiveness in mate selection. For instance, individuals who are currently dating tend to invest more time in beauty enhancement compared to those in established relationships (Kowal et al. 2022). Moreover, marrying a wealthy man continues to be a viable way for women to approach economic goals (Chen 2018).

Attractiveness can be advantageous not only in personal life but also in the labor market, where a ‘pretty face’ is often perceived as beneficial for women in professional settings (Wen 2013). Empirical studies have consistently shown that beauty positively impacts workplace outcomes. For example, more attractive individuals tend to earn higher salaries both early in their careers (Dossinger et al. 2019) and throughout their lifetimes (Scholz and Sicinski 2015). Makeup can enhance women’s perceived leadership abilities when applying for jobs and contribute to creating favorable impressions in the workplace (Blake 2022; Netchaeva and Rees 2016). The beauty premium is evident even before the interview stage (Deng et al. 2020). More recently, Wang et al. (2022) found that in competitive environments, women are more likely to invest in their appearance, particularly those who believe that sex equates to power.

### Empowerment or oppression?

Gill (2007) introduced the concept of “postfeminist sensibility” as a flexible framework for analyzing how popular media culture portrays women as women as self-made, savvy, and empowered consumers. Within this perspective, beauty practices are often framed as personal choices that signify empowerment across various global contexts (Riley et al. 2017). As a part of this discourse, beauty is increasingly recognized as a form of social and economic capital, a notion that has gained significant traction in recent decades (Gill 2008). Women, particularly those from middle- and upper-class backgrounds, often embrace postfeminist ideals of empowered femininity, self-transformation, and agency, strategically leveraging their physical appearance as a form of capital (Genz 2015).

However, the perception of attractiveness as a tool for empowerment is deeply embedded in broader power dynamics and gender norms. Butler (1988) argued that sex is not merely a biological fact but a dynamic process in which regulatory norms continuously materialize and reinforce gendered performances. These norms eroticize relationships between men and women, reinforcing the social desirability of masculinity and femininity while maintaining existing power structures. In this context, women are often encouraged to view sexual empowerment as a pathway to liberation, believing that control over their appearance translates into personal and social power (Couture Bue and Harrison 2019). Consequently, attractiveness is framed as a form of agency dictated by prevailing social norms. As a result, some women internalize an external observer’s perspective, prioritizing their physical appearance over their competence (Calogero 2013a).

For women facing gender-based earnings disparities, enhancing their appearance may seem to offer a sense of control and empowerment. Empirical evidence supports this idea, showing that some women report feeling more powerful due to their attractiveness. For instance, self-objectified women have experienced an increased sense of power when being observed (Breines et al. 2008). Similarly, women who engage in self-sexualizing behaviors often interpret these actions as expressions of sexual empowerment (Donaghue et al. 2011). More recently, studies have found that women who use makeup report higher levels of psychological assertiveness, particularly in romantic contexts (Blake et al. 2020).

However, this perceived empowerment may be superficial or even illusory. The reinforcement of gendered performances naturalizes gender differences, presenting them as inevitable and even desirable, thereby perpetuating existing inequalities (Butler 1988). Some scholars argue that an appearance-focused culture dehumanizes women, making self-objectification a mechanism of control rather than genuine empowerment (Choi and DeLong 2019; De Wilde et al. 2021; Gill 2008). Empirical evidence supports this claim, showing that self-objectification functions more as a form of oppression than liberation (Choi and DeLong 2019; De Wilde et al. 2021). Studies indicate that sexualized women are more likely to be objectified than those who do not present themselves in a sexualized manner (Cogoni et al. 2023; Vaes et al. 2019). Moreover, excessive concerns with appearance are often associated with perceptions of diminished competence, warmth, morality, and even humanity (e.g., Bernard et al. 2020; Kellie et al. 2019). Female college students, for example, have been found to impose a “strategic beautification penalty” on peers who use cosmetics to enhance their appearance, expressing less interest in interacting with them (Delpriore et al., 2018).

In summary, a clear discrepancy exists between how women perceive the effects of prioritizing their appearance and how third-party observers interpret these actions. While women may view attractiveness as a source of empowerment, external observers may instead see it as self-dehumanization. Specifically, women who prioritize their appearance may be regarded as less human, less empowered, and more vulnerable to objectification in daily life. Additionally, individuals may be less willing to form friendships with highly attractive women. These effects are likely to be observed among both men and women.

### The present research

Based on the preceding theoretical and empirical evidence, we formulated the following hypotheses. First, we propose that women in the context of gender earnings inequality context perceive empowerment from attractiveness (H1). In contrast, third-party observers perceive women who emphasize attractiveness as dehumanized in the same context (H2). To test these predictions, we conducted two studies. Study 1 examined women’s perceptions of attractiveness when encountering the gender earnings inequality. Study 2 investigated third-person perspectives on physical appearance in the context of gender earnings inequality.

For sample size determination, in Study 1, we aimed for 100 participants per condition. Priori sample size analyses using G*Power (Faul et al. 2007) were conducted in Study 2. Based on previous studies on the effect of macroeconomic factors on individuals’ mind and behavior (e.g., Cheng et al. 2020), we set a small to medium effect size (*d* = 0.35). Sensitivity power analyses indicated that minimum detectable effect sizes were *f* = 0.20 for Study 1 (*N* = 200) and *f* = 0.19 for Study 2 (*N* = 219), under standard criteria (*α* = 0.05 two-tailed, *β* = 0.80). These sample sizes were sufficient to detect small-to-medium effects (*f* = 0.10–0.25). The actual effect sizes found across our studies fell within the small-to-medium range (Study 1: η_p_^2^ = 0.017, Study 2: η_p_^2^ = 0.03). Study 1 was preregistered (https://aspredicted.org/9W5_W6J). Since women’s focus on attractiveness is linked to age, Body Mass Index (BMI, calculated from weight and height), education, and socioeconomic class (Jackson and Chen 2008; Labunskaya 2019; Rojo-Ramos et al. 2023), and income and socioeconomic class may relate to perceived gender earnings inequality, we controlled for key demographics, including age, education, income, subjective socioeconomic class (SSS; Adler et al. 2000), and BMI. We reported results with covariates in the text and results without covariates in the supplementary material.

### Study 1

Study 1 aimed to investigate women’s perceptions of attractiveness in the context of gender earnings inequality. We expected that women experiencing gender earnings inequality would perceive attractiveness as a form of empowerment in both dating and job interview settings.

## Methods

### Participants

Two hundred heterosexual Chinese women (*M*_*age*_ = 23.25 years, *SD* = 3.85) were recruited from Weidiaocha (www.weidiaocha.cn), a Chinese participants recruitment platform comparable to Prolific. One hundred and three participants were assigned to the gender earnings inequality condition, and 97 were assigned to the gender earnings equality condition. All participants gave informed consent prior to their participation and received a small amount of compensation at the end of the study.

### Procedure

The gender earnings inequality was manipulated by asking participants to imagine that they were going to emigrate to another country called Bimboola (Sánchez-Rodríguez et al. 2019), where residents’ gender earnings inequality was categorized into one of the two levels. In the gender earnings inequality condition, participants were informed that the average monthly income of single men is 17,500 BD (the monetary unit of Bimboola) and that of women is 7,000 BD. Additionally, men possess 72% of the total wealth, and women possess 28% (i.e., men own 2.57 times more wealth than women). In the control condition, the participants were told that Bimboola has gender earnings equality, with both men and women earning an average of 7,000 BD a month and each possessing 50% of the total wealth in society. To strengthen the gender earnings inequality manipulation, participants were then asked to purchase three necessities (a house, car, and holiday) they could afford from a set of options. The options available to men in the gender earnings inequality condition were more luxurious than those available to women, while the options available to men in the low gender earnings inequality condition were identical to those available to women. As a member of women, participants were able to choose from the items available to women, but they could not afford any options available to men.

To determine whether the gender earnings inequality manipulation was effective, participants were asked the following question: “In Bimboola, to what extent is the gender income distribution unequal? (1 *= not at all*, 9 *= very much*).”

Next, participants were asked to think about their desired job and heterosexual men. They were then asked to imagine they were interviewing for a job and going on a date with a man, and to report their perceived benefit from their attractiveness in both scenarios. Afterward, participants completed a measure of the belief to explore the potential mediation effect.

Finally, participants completed a measure of control variables, including identification with Bimboola and affect, to account for potential influences of negative affect induced by the gender pay inequality manipulation. They also provided demographic information.

### Measures

*Perceived benefit from attractive appearance.* Five items were used to measure their perception of benefit through attractiveness in both the interview and dating contexts. The items were: “In Bimboola, an attractive appearance helps me get an offer/attract a man,” “In Bimboola, attractive appearance is an important factor for me to successfully get an offer/attract a man,” “In Bimboola, attractiveness helps me make a good impression on the interviewer/the man,” “In Bimboola, attractiveness helps me control the interview/dating,” and “In Bimboola, attractiveness helps me get the upper hand in the interview/dating.” Participants rated their agreement with each statement on a scale from 1 (*completely disagree*) to 7 (*completely agree*) (*α*_interview_ = 0.87; *α*_dating_ = 0.85).

*Beauty is power belief.* Participants completed an 8-item scale (adapted from Erchull and Liss 2013) and rated their agreement with each statement on a scale from 1 (*completely disagree*) to 7 (*completely agree*). Example items included: “In Bimboola, most women can use their beauty to achieve social mobility,” and “In Bimboola, beauty is sometimes a resource that women can leverage” (*α* = 0.89).

### Control variables

*Identification with Bimboola.* Two items were used to measure the participants’ identification with Bimboola. The items were “To what extent do you identify as a member of Bimboola society?” and “I can easily imagine myself as a member of the Bimboola society.” (*1 = Strongly disagree*, *9 = Strongly agree*). The average score was calculated to represent the scale of identification with Bimboola society (*M* = 4.31, *SD* = 2.02; *α* = 0.82).

*Affect.* The Positive and Negative Affect Schedule Short Form (PANAS-SF; Thompson 2007) was used to measure participants’ affects. The scale consists of 10 affects: 5 positive affects (reversed; PA: active, determined, attentive, inspired, and alert) and 5 negative affects (NA: afraid, nervous, upset, hostile, and ashamed). Participants were asked to rate each affect on a 5-point scale (*1 = Not at all*, *5 = Extremely*). A higher average score on the scale indicates stronger negative affect (*α*_*PA*_ = 0.72, *α*_*NA*_ = 0.77).

Demographic information, including age, education level, annual household income, and SSS (Adler et al. 2000) were also collected. Additionally, participants reported their weight and height, which were used to calculate BMI.

## Results and discussion

### Manipulation check

An independent-samples *t* test revealed that participants in the gender earnings inequality condition (*M* = 7.43, *SD* = 1.92) reported a higher earnings inequality between women and men than those in the control condition (*M* = 2.05, *SD* = 1.67), *t*(198) = 21.13, *p* < .001, Cohen’s *d* = 2.99, indicating that the manipulation was valid.

### Perceived benefit of attractiveness

Using ANCOVAs (control variables: age, BMI, education level, annual household income, subjective social class, identity, and affects), we found that in the dating context, participants in the gender earnings inequality condition (*M* = 5.17, *SD* = 1.11) perceived higher benefit than those in the control condition (*M* = 4.96, *SD* = 1.07), *F*(1, 190) = 4.80, *p* = .03, η_p_^2^ = 0.025. Additionally, in the job interview context, participants in the gender earnings inequality condition (*M* = 4.11, *SD* = 1.31) reported marginally higher benefit than those in the control condition (*M* = 4.01, *SD* = 1.26), *F*(1, 190) = 3.28, *p* = .07, η_p_^2^ = 0.017.

Furthermore, participants in the gender earnings inequality condition (*M* = 3.70, *SD* = 1.24) reported a higher belief in the idea that beauty is power than those in the control condition (*M* = 3.34, *SD* = 1.23), *F*(1, 190) = 12.35, *p* < .001, η_p_^2^ = 0.06.

### Exploratory mediation effect of beauty is power belief

**Fig. 1:**
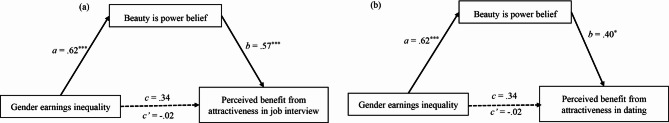
Beauty is power belief mediate the effect of gender earnings inequality on benefit from attractiveness in job interview (**a**) and dating (**b**) in Study 1. *Note.* Unstandardized coefficients are displayed. The c path shows the total effect and the c’ path shows the direct effect. ^***^p < .001

We conducted bootstrapping mediation analyses with 5000 iterations (Model 4; Hayes 2013). Specifically, we modeled the condition (gender earnings inequality versus control) as the independent variable, beauty is power belief as the mediator, and the perceived benefit of beauty in the interview and dating as dependent variables. Control variables included age, BMI, education level, annual household income, subjective social class, identity, and affects (see Fig. 1).

The results showed that the indirect effect of “beauty is power” belief on the relationship between the gender earnings inequality and the perceived benefit of attractiveness were significant in both the interview (*b* = 0.35, *SE* = 0.10, 95% CI [0.16, 0.56]) and the dating context (*b* = 0.25, *SE* = 0.07, 95% CI [0.12, 0.40]). Specifically, when predicting the perceived benefit of attractiveness in interviews, gender earnings inequality significantly predicted the “beauty is power” belief (*b* = 0.62, *SE* = 0.18, *p* = .001, 95% CI [0.27, 0.97]). Additionally, controlling for gender earnings inequality, the “beauty is power” belief significantly predicted the perceived benefit of attractiveness in interviews (*b* = 0.57, *SE* = 0.06, *p* < .001, 95% CI [0.44, 0.70]; see Fig. 1a). Similarly, when predicting the perceived benefit of attractiveness in dating, gender earnings inequality significantly predicted the “beauty is power” belief (*b* = 0.62, *SE* = 0.18, *p* = .001, 95% CI [0.27, 0.97]). Additionally, controlling for gender earnings inequality, the “beauty is power” belief significantly predicted the perceived benefit of attractiveness in dating (*b* = 0.40, *SE* = 0.06, *p* < .001, 95% CI [0.28, 0.51]; see Fig. 1b).

From a first-person perspective, we examined the perceived benefit of attractiveness among women who experienced a gender earnings inequality. Our findings indicate that women reported feeling greater benefit from their attractiveness when facing gender-based earnings disparities. Additionally, the perceived benefit of attractiveness was stronger in dating contexts than in job interviews. This may be due to the greater emphasis on competence rather than appearance in the workplace, whereas in mating contexts, attractiveness is valued more (Davis and Arnocky 2022). However, it remains unclear whether individuals, from a third-person perspective, perceive the benefit of attractiveness in the same way as women experiencing the gender earnings gap. To address this question, Study 2 examined third-person perceptions of women’s appearance concern.

### Study 2

Study 2 aimed to explore whether third-party observers perceive appearance concern as a form of empowerment for women facing gender earnings inequality. We hypothesized that, contrary to the empowerment perspective, individuals would actually view appearance concern as a form of self-dehumanization for these women. Consequently, we anticipated that these women would be rated as less human, less competent, and more likely to be objectified in daily life, with third-party observers showing less interest in befriending them. We further predicted that this effect would be consistent across both male and female participants. To test this hypothesis, we included participants of both genders in the present study.

## Methods

### Participants

An a priori sample size analysis for a two-factor between-subjects ANOVA showed that at least 219 participants were needed to detect a small-to-medium effect size (η_*p*_^2^ = 0.035), with a statistical power of 80% and an alpha level of 0.05 (Faul et al. 2007). A total of 219 heterosexual Chinese participants (*M*_*age*_ = 27.17 years, *SD* = 6.04; men, 51.1%) were recruited from Weidiaocha. All participants gave informed consent prior to their participation and received a small amount of compensation at the end of the study.

### Procedure

The participants were informed that the study aimed to examine the effect of impression management. To manipulate the gender earnings inequality, participants were shown three pie charts depicting the wealth distributions between men and women in three anonymous countries (Countries *M*, *L*, and *K*; Heiserman and Simpson 2017). To avoid making assumptions about other features of the three countries, participants were informed that the countries have similar GDPs per capita, political systems, religious beliefs and levels of economic inequality. Participants were then told that they would answer questions about Country *M*, which, depending on the condition, was described as having either a high or low gender earnings inequality relative to the other two countries. In country M/K (with high gender earnings inequality), men account for 85% of the average salary, while women account for 15%. In country L (with a moderate gender earnings inequality), men account for 72% of the average salary, while women account for 28%. In country K/M (control condition, with low gender earnings inequality), men receive 52% of the total pay, while women receive 48%. However, in the present study, all participants were assigned to the gender earnings inequality condition.

To check participants’ comprehension, they completed two manipulation check items: “Which country has the most unequal distribution of pay by gender?” and “Which country has the most equal distribution of pay by gender?” (1 *= M*, 2 *= L*, 3 *= K*). Only participants who answered these questions correctly were allowed to proceed with the study.

Next, participants were randomly assigned to either the appearance concern condition (*n* = 119, 47.9% female) or the control condition (*n* = 100, 50% female; adapted from De Wilde et al. 2021; Study 2). The participants were shown a *Body Attitude Scale* consisting of 10 items (adopted from Calogero et al. 2017; McKinley and Hyde 1996; e.g., “I often worry about whether the clothes I am wearing make me look good.” 1 *= completely disagree*, 7 *= completely agree*), completed by a woman named *Ms. A* who lives in country *M*. In the appearance concern condition, participants were shown that *Ms. A* was more concerned about her appearance than her competence (i.e., with 5 items rated as *agree*, and another 5 items as *completely agree*). In the control condition, participants were shown that *Ms. A* was more concerned about her competence than her appearance, i.e., with less self-objectification (i.e., with 5 items rated as *disagree* and another 5 items rated as *completely disagree*). The manipulation check was assessed using a single item: “To what extent do you think *Ms. A* is concerned about their appearance?” (1 = *not at all*, 7 = *extremely*). To enhance the manipulation effect, participants were instructed to write a short essay about their thoughts on *Ms. A*.

Next, participants completed the measures of the dependent variables, including the target’s humanness, perceived empowerment of the target, likelihood of sexual objectification in daily life, and intentions to befriend the target. Finally, participants reported their demographic information and affect, identical to Study 1, before being thanked and debriefed. However, we did not collect participants’ identification with the country in Study 2, as the manipulation of gender earnings inequality was not suitable for measuring identification.

### Measures

*Humanness.* Sixteen attributes were used to rate the humanness of the target across four aspects: agency (assertive, independent, ambitious, and determined), warmth (warm, friendly, sociable, and likeable), competence (competent, clever, efficient, and capable), and morality (honest, sincere, trustworthy, and righteous) (De Wilde et al. 2021). Participants were asked, “To what extent do you perceive the woman as having the following attributes?” Each aspect was measured with 4 items (1 *= Completely disagree*, 7 *= Completely agree*). The average score was calculated, with a higher score indicating greater perceived humanness of the target (*α* = 0.94).

*Perceived empowerment of the target.* Participant’s perceived empowerment of the target was measured using seven items adapted from Kim et al. (2018). An example item was “In country *M*, is *Ms. A* in control of her life?” (1 *= Strongly disagree*, 7 *= Strongly agree*). The average score was used as an indicator of perceived empowerment, with a higher score reflecting a higher degree of perceived empowerment (*α* = 0.92).

*Likelihood of sexual objectification in daily life.* The Interpersonal Sexual Objectification Scale (Kozee and Tylka 2007) was used to measure the perceived likelihood of sexual objectification of the target in daily life. The participants rated 15 items assessing the frequency of sexual objectification the target encountered in daily life while living in country *M* using a 7-point scale (e.g., “being touched or caressed against her will”; 1 *= Never*, 7 *= All the time*). The average score was calculated as an indicator of the likelihood of objectification, with a higher score indicating a greater likelihood of being objectified (*α* = 0.98).

*Intentions to befriend the target.* Four items were used to measure participants’ intentions to befriend the target (e.g., “To what extent do you want to make friends with *Ms. A*?” 1 *= Not at all*, 7 *= Very much*). The average score was calculated, with a higher score indicating a stronger intention to befriend the target (*α* = 0.91).

*Control variables.* The PANAS-SF (*α*_*PA*_ = 0.66, *α*_*NA*_ = 0.80) and demographic information were collected as in Study 1.

## Results and discussion

### Manipulation check

The independent-samples *t* test showed that the participants in the appearance concern condition (*M* = 6.74, *SD* = 0.60) reported significantly higher self-objectification than those in the control condition (*M* = 2.05, *SD* = 1.27), *t*(217) = 35.68, *p* < .001, 95% CI [4.43, 4.95], Cohen’s *d* = 4.84, indicating a successful manipulation.

### Participants’ perceptions of the target’s concern with appearance

To examine the perceived effect of attractiveness in the context of gender earnings inequality, a between-participants MANOVA was conducted to assess the extent to which appearance concern (control condition = 0, appearance concern condition = 1) and participant gender (male = 0, female = 1) influenced the dependent variables (humanness, empowerment, likelihood of objectification, and intention to form friendships). Demographic variables and affect were controlled as covariates. The multivariate test revealed a significant main effect of appearance concern, *λ* = 0.61, *F*(4, 207) = 33.65, *p* < .001, η_*p*_^2^ = 0.394.

**Fig. 2:**
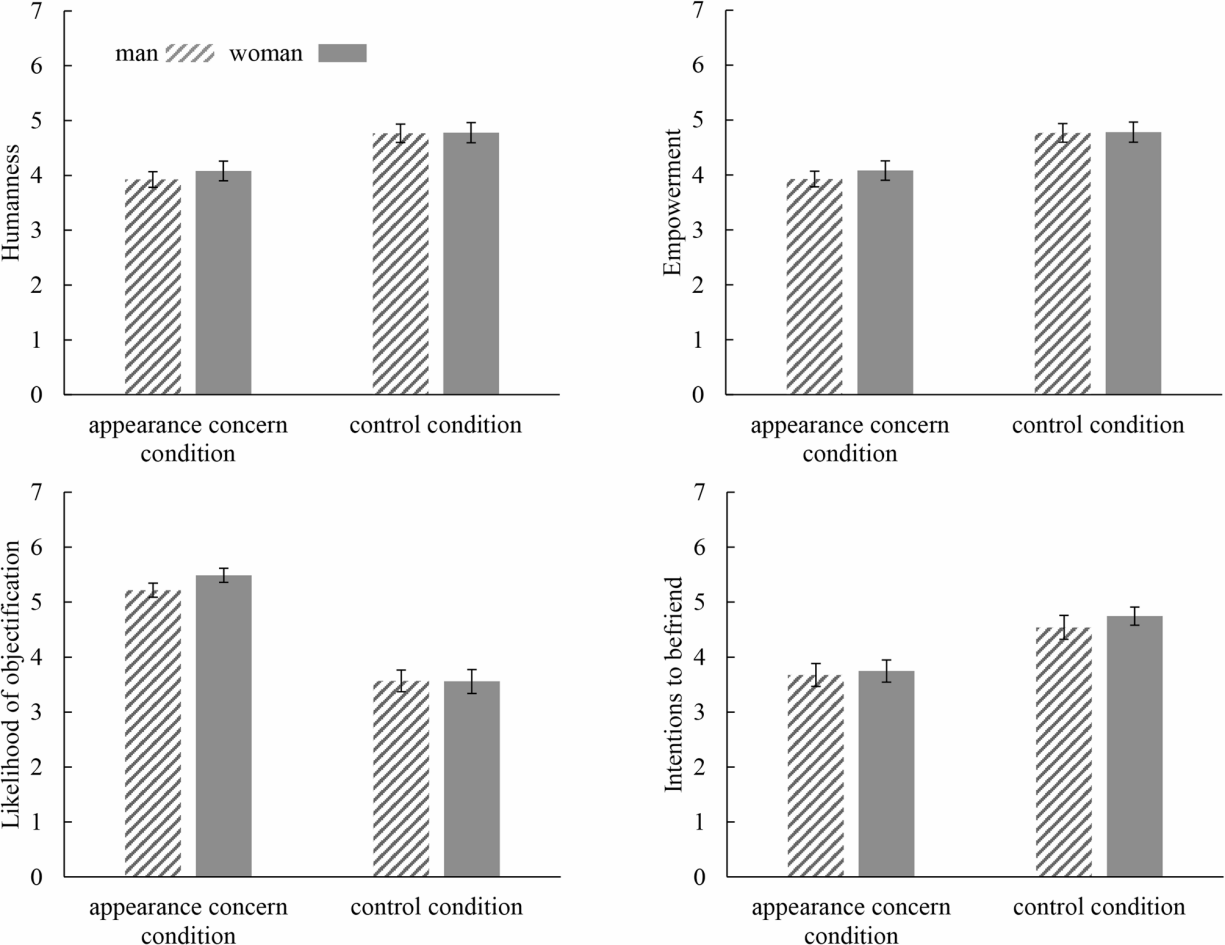
Participants’ perceptions of the target’s concern with appearance

It was demonstrated that neither the main effect of the participant’s gender nor the interaction effect reached significance when predicting humanness, empowerment, the likelihood of objectification, or the intention of making friends (*p*s > 0.50), while the main effect of appearance concern was significant. Specifically, participants in the appearance concern condition rated the target as less human (*F*(1, 210) = 24.24, *p* < .001, η_p_^2^ = 0.10; *M*_*appearance concern condition*_ = 4.00, *SD* = 1.23; *M*_*control condition*_ = 4.77, *SD* = 1.24), less empowered (*F*(1, 210) *=* 5.81, *p* = .02, η_p_^2^ *=* 0.03; *M*_*appearance concern condition*_ = 3.58, *SD* = 1.22; *M*_*control condition*_ = 4.06, *SD* = 1.60), and more likely to encounter objectification in daily life (*F*(1, 210) *=* 104.32, *p* < .001, η_p_^2^ *=* 0.33; *M*_*appearance concern condition*_ = 5.35, *SD* = 0.99; *M*_*control condition*_ = 3.56, *SD* = 1.47), as well as less likely to form friendships with the target (*F*(1, 210) *=* 20.50, *p* < .001, η_p_^2^ *=* 0.089; *M*_*appearance concern condition*_ = 3.71, *SD* = 1.41; *M*_*control condition*_ = 4.64, *SD* = 1.51; Fig. 2).

Error bars represent the standard error around the mean.

From a third-person perspective, we observed that women who prioritize an attractive appearance in the context of gender earnings inequality are perceived as less human, less empowered, more likely to experience sexual objectification in daily life, and less likely to be chosen as friends. Additionally, both male and female observers tend to perceive these women as dehumanized and alienated due to their emphasis on appearance. It is possible that cues related to a woman’s concern with her appearance are so prominent that both genders judge her primarily based on her physical appearance rather than her personality or competence.

## General discussion

In this research, we conducted two studies to examine how women’s attractiveness is perceived in the context of gender earnings inequality, drawing on both first-person and third-person perspectives. Study 1 explored women’s own experiences and revealed that when faced with gender earnings inequality, women reported feeling more empowered when encouraged to focus on their appearance—supporting H1. In contrast, Study 2 adopted a third-person perspective and found that both male and female observers perceived women who emphasized their attractiveness in such a context as more dehumanized and socially alienated—supporting Hypothesis 2. Together, these findings underscore a striking discrepancy: while women may personally experience a sense of empowerment through leveraging attractiveness in unequal gender contexts, external observers tend to view this strategy in a more negative, objectifying light.

### Theoretical and practical implications

Our study contributes to the growing literature on the antecedents of beautification by examining women’s experiences within the context of gender-based economic disadvantage, specifically gender earnings inequality. From a functional perspective, attractiveness may serve two key roles: aiding women in competing for high-quality genetic partners (Davis and Arnocky 2022) and securing reproduction-related resources, such as social status and career opportunities (Blake 2022). Consequently, women’s concern for their appearance may stem from these adaptive functions. Our findings provide preliminary evidence for integrating evolutionary and sociocultural perspectives on attractiveness, shedding light on both its functions and underlying antecedents (e.g., Blake 2022; Davis and Arnocky 2022).

Beyond its functional role, our study extends previous research on self-objectification by considering its potential adaptive aspects. While prior studies have extensively documented the negative physical and mental health consequences of self-objectification (Roberts et al. 2018), not all appearance concerns lead to detrimental outcomes, nor are individuals merely passive recipients of their social norms (e.g., Breines et al. 2008). In economically disadvantaged contexts, attractiveness may function as a strategic tool for women to navigate social constraints. That is, when traditional pathways to social mobility are limited, beautification can serve as a means of empowerment. However, self-objectification is shaped by multiple intersecting factors, including social, cultural, and economic influences. As women’s bodies can simultaneously be sites of empowerment and oppression (Gill 2007), it is crucial to move beyond a simplistic, dichotomous view of appearance concerns as inherently positive or native, moral or immoral (Wen 2013).

Our findings contribute to the literature on postfeminism by examining both first-person and external perspectives on women’s attractiveness. Gill (2007) highlights that a defining feature of postfeminist media is its obsessive focus on the female body, portraying it as both a source of power and an unruly entity requiring constant surveillance, discipline and modification to meet increasingly rigid beauty standards. While women may perceive attractiveness as empowering, this sense of empowerment is often fleeting, superficial and illusory (Bartky 1990; Fredrickson and Roberts 1997; Gill 2008; Jeffreys 2005; Wolf 1990). This illusion can contribute to the depressed-entitlement effect (Calogero 2013a), reinforcing women’s acceptance of systemic inequalities.

Moreover, prioritizing appearance can lead to unintended negative consequences, such as increased vulnerability to dehumanizing treatment in interpersonal relationships and a decreased likelihood of being chosen as a friend in the present study. Postfeminist discourses further entrench the existing inequalities by normalizing and sustaining the broader gender order (Gill 2007). This supports the argument that self-objectification is not merely an individual concern but part of a larger system-justifying process that perpetuates gender inequality by maintaining women’s dehumanized status (Calogero 2013b). Consequently, while attractiveness may provide short-term benefits, the long-term social and psychological costs of being perceived as attractive may ultimately outweigh these advantages.

### Limitations and future directions

Despite the contributions of this study, there are some limitations that should be noted, and directions for future research that should be raised. First, while our findings suggest that women perceive attractiveness as relevant to navigating gender earnings inequality, it remains unclear whether attractiveness translates into real power. Our aim was to capture women’s perceived empowerment through female attractiveness; however, we acknowledge that the phrasing may not fully clarify whether attractiveness actually translates into real power. Future research is encouraged to further explore the extent to which attractiveness can help women generate both tangible and intangible advantages.

Moreover, although our findings from Study 1 indicate that women in the gender earnings inequality condition perceived attractiveness as increasingly important, the underlying psychological mechanism remains warrants further investigation. Women frequently engage in self-presentation through beautification (Wen 2013); however, our study specifically highlights how the socioeconomic environment—particularly gender earnings inequality—in amplifying the instrumental value of attractiveness, similar to the increased lipstick consumption observed during economic recessions (Hill et al. 2012; Netchaeva and Rees 2016). In other words, economic disparities may heighten women’s awareness of their appearance can serve as a form of social and economic capital. Future research should further examine the motivations driving women’s pursuit of attractiveness, particularly in response to structural inequalities.

Second, this study exclusively recruited Chinese women, which may limit the generalizability of our findings. While women can pursue economic equality through both employment and partnerships, cultural differences may shape the extent to which beauty influences these pathways. East Asian cultures tend to prioritize interpersonal relationships, whereas Western cultures place greater emphasize on individual independence and agency (Hofstede 2001). Consequently, Eastern women may be more inclined to pursue economic goals through mating strategies, whereas Western women may be more likely to compete in the labor market. Future research should include participants from both Eastern and Western cultural backgrounds to provide a more comprehensive perspective. Additionally, our sample consisted of young women (mean age: 23.25 years in Study 1 and 27.17 years in Study 2), which may limit the generalizability of our findings to older women. This limitation is particularly relevant given that attractiveness concerns and self-objectification tend to be more pronounced during emerging adulthood (Fredrickson and Roberts 1997). Future research is encouraged to examine the role of age in shaping women’s perceptions of attractiveness and its associated implications.

Third, our study focused on “perceived empowerment” from attractiveness, defined as the belief that one’s attractiveness yield social or professional advantages. Unlike traditional empowerment, which emphasizes control and agency, our measurements capture perceived benefits rather than direct agency. Since attractiveness is often externally judged trait and not always be under an individual’s control, one may perceive attractiveness as influential while lacking personal agency over it. Future research should further distinguish between perceived benefits and actual agency by assessing individuals’ perceived control over their attractiveness (e.g., self-efficacy in enhancing or leveraging attractiveness). This could provide a more comprehensive understanding of empowerment.

Additionally, regarding the alignment between Study 1 and Study 2, the measures used in the two studies are not directly comparable. Study 1 focused on how women perceive the benefits of attractiveness, whereas Study 2 examined how external observers evaluate women’s concerns about their appearance. Our primary goal was to explore how third-party observers interpret women’s appearance concerns in a gendered context, rather than to replicate the measures of Study 1. Furthermore, we did not assess self-alienation or self-dehumanization in Study 2. Future research could benefit from incorporating these constructs to provide a more comprehensive understanding of how attractiveness influences women’s agency and societal treatment.

Fourth, although attractiveness may help address gender earnings inequality, its long-term effectiveness is limited. A critical question is whether women are aware of the gender oppression inherent in cultural beauty ideals. Evidence from research on benevolent sexism suggests that women may be encouraged to engage in self-objectification and body surveillance by seemingly positive messages (Hopkins-Doyle et al. 2019). Similarly, women who experience the gender earnings inequality may be more likely to focus on short-term benefits and may not have yet recognized the underlying sexual oppression of embedded in cultural ideals of beauty (Calogero 2013a). While economic disadvantages may lead women to prioritize short-term benefits rather over long-term rewards, this hypothesis requires further investigation from a cognitive psychology perspective.

Finally, we adopted a 7:3 male-to-female wealth ratio, meaning that men own 2.57 times more wealth than women. While this ratio may seem stark, it was deliberately chosen to reflect significant gender earnings disparities. Our decision was informed by the Global Gender Gap Report (World Economic Forum 2024), which reported that the global gender gap in the Economic Participation and Opportunity subindex is 60.5%. This indicates that if men score 1.0 in this subindex, women score 0.605, resulting in a male-to-female ratio of 1.65. To highlight the implications of gender earnings inequality, we intentionally exaggerated this ratio in our study. The 7:3 ratio was also selected for its simplicity, ensuring that participants could easily comprehend the disparity. Future research should further explore how different levels of gender earnings inequality influence women’s perceptions of attractiveness.

## Conclusion

We explored perceptions of attractiveness from both first-person and third-person perspectives. Our findings suggest that women themselves may perceive attractiveness as a source of empowerment in response to gender earnings inequality. However, from the perspective of external observers, appearance concerns can lead to perceptions of dehumanization, contributing to a sense of alienation.

## Supplementary Information

Below is the link to the electronic supplementary material.

Supplementary Material 1

## Data Availability

The dataset(s) supporting the conclusions of this article are available in the OSF repository, unique persistent identifier and hyperlink to dataset(s) in https://osf.io/rkwxn/?view_only=6442c9ad78f14cacaf6d5bedc5e8d01a.

## Abbreviations

M: Mean
SD: Standard deviation
CI: Confidence interval

## References

[CR1] Adler NE, Epel ES, Castellazzo G, Ickovics JR. Relationship of subjective and objective social status with psychological and physiological functioning: preliminary data in healthy, white women. Health Psychol. 2000;19(6):586–92. 11129362 10.1037//0278-6133.19.6.586

[CR2] Bartky SL. Femininity and domination: studies in the phenomenology of repression. New York: Routledge; 1990.

[CR3] Baumeister RF, Vohs KD. Sexual economics: sex as female resource for social exchange in heterosexual interactions. Personality Social Psychol Rev. 2004;8(4):339–63. 10.1207/s15327957pspr0804_2 15582858

[CR4] Bernard P, Content J, Servais L, Wollast R, Gervais S. An initial test of the cosmetics dehumanization hypothesis: heavy makeup diminishes attributions of humanness-related traits to women. Sex Roles. 2020;83:315–27.

[CR5] Bertrand M, Goldin C, Katz LF. Dynamics of the gender gap for young professionals in the financial and corporate sectors. Am Economic Journal: Appl Econ. 2010;2(3):228–55.

[CR6] Blake KR. Attractiveness helps women secure mates, but also status and reproductively relevant resources. Arch Sex Behav. 2022;51(1):39–41. 33666826 10.1007/s10508-021-01949-2

[CR7] Blake KR, Brooks R, Arthur LC, Denson TF. (2020). In the context of romantic attraction, beautification can increase assertiveness in women. PLoS ONE. 15(3):e0229162. 10.1371/journal.pone.0229162 10.1371/journal.pone.0229162 PMC7064170 32155157

[CR8] Blau FD, Kahn LM. Understanding international differences in the. Gender earnings gap. J Labor Econ. 2003;21(1):106–44.

[CR9] Breines JG, Crocker J, Garcia JA. Self-objectification and well-being in women’s daily lives. Pers Soc Psychol Bull. 2008;34:583–98. 18281441 10.1177/0146167207313727

[CR10] Buss DM. Sex differences in human mate preferences: evolutionary hypotheses tested in 37 cultures. Behav Brain Sci. 1989;12(01):1–14.

[CR11] Butler J. Performative acts and gender constitution: an essay in phenomenology and feminist theory. Theatre J. 1988;40(4):519–31. 10.2307/3207893.

[CR12] Calogero RM. (2013a). On objects and actions: Situating self-objectification in a system justification context. In S. Gervais, editor, *Nebraska motivation symposium: Vol. 60. Perspectives on motivation* (pp. 97–126). New York, NY: Springer. 10.1007/978-1-4614-6959-9_5 23947280

[CR13] Calogero RM. Objects don’t object: evidence that self-objectification disrupts women’s social activism. Psychol Sci. 2013b;24(3):312–8. 23341162 10.1177/0956797612452574

[CR14] Calogero RM, Tylka TL, Donnelly LC, McGetrick A, Leger AM. Trappings of femininity: A test of the beauty as currency hypothesis in shaping college women’s gender activism. Body Image. 2017;21:66–70. 28315810 10.1016/j.bodyim.2017.02.008

[CR15] Chen M. Does marrying well count more than career? Personal achievement, marriage, and happiness of married women in urban China. Chin Sociol Rev. 2018;50(3):240–74.

[CR16] Cheng L, Zhou X, Wang F, Hao M. The greater the economic inequality, the later people have children: the association between economic inequality and reproductive timing. Scand J Psychol. 2020;61(3):450–9. 32012300 10.1111/sjop.12612

[CR17] Choi D, DeLong M. Defining female self sexualization for the twenty-first century. Sex Cult. 2019;23(4):1350–71.

[CR18] Cogoni C, Monachesi B, Mazza V, Grecucci A, Vaes J. (2023). Neural dynamics of vicarious physical pain processing reflect impaired empathy toward sexually objectified versus non-sexually objectified women. Psychophysiology, 60(12), e14400. 10.1111/psyp.14400 10.1111/psyp.14400 37578333

[CR19] Couture Bue AC, Harrison K. Empowerment sold separately: two experiments examine the effects of ostensibly empowering beauty advertisements on women’s empowerment and self-objectification. Sex Roles. 2019;81(9):627–42.

[CR20] Davis AC, Arnocky S. An evolutionary perspective on appearance enhancement behavior. Arch Sex Behav. 2022;51(1):3–37. 33025291 10.1007/s10508-020-01745-4

[CR22] De Wilde M, Carrier A, Casini A, Demoulin S. The drawback of sexual empowerment: perceiving women as emancipated but still as sexual objects. Sex Roles. 2021;84(9):626–43.

[CR23] DelPriore DJ, Bradshaw HK, Hill SE. Appearance enhancement produces a strategic beautification penalty among women. Evolutionary Behav Sci. 2018;12(4):348–66.

[CR21] Deng W, Li D, Zhou D. Beauty and job accessibility: new evidence from a field experiment. J Popul Econ. 2020;33:1303–41.

[CR24] Donaghue N, Kurz T, Whitehead K. Spinning the pole: A discursive analysis of the websites of recreational pole dancing studios. Feminism Psychol. 2011;21:443–57.

[CR25] Dossinger K, Wanberg CR, Choi Y, Leslie LM. The beauty premium: the role of organizational sponsorship in the relationship between physical attractiveness and early career salaries. J Vocat Behav. 2019;112:109–21.

[CR26] Erchull MJ, Liss M. Exploring the concept of perceived female sexual empowerment: development and validation of the sex is power scale. Gend Issues. 2013;30(1–4):39–53.

[CR27] Erchull MJ, Liss M. The object of one’s desire: how perceived sexual empowerment through objectification is related to sexual outcomes. J Sexuality Cult Med Psychiatry. 2014;18(4):773–88.

[CR28] Faul F, Erdfelder E, Lang AG, Buchner A. G*Power 3: A flexible statistical power analysis program for the social, behavioral, and biomedical sciences. Behav Res Methods. 2007;39(2):175–91. 17695343 10.3758/bf03193146

[CR29] Fiske ST, Berdahl JL. Social power. In: Higgins ET, Kruglanski AW, editors. Social psychology: handbook of basic principles. 2nd ed. London, England: Oxford University Press; 2007. pp. 678–92.

[CR30] Fredrickson BL, Roberts TA. Objectification theory: toward Understanding women’s lived experiences and mental health risks. Psychol Women Q. 1997;21(2):173–206.

[CR31] Genz S. My job is me: postfeminist celebrity culture and the gendering of authenticity. Feminist Media Stud. 2015;15(4):545–61.

[CR33] Gill R. Postfeminist media culture: elements of a sensibility. Eur J Cult Stud. 2007;10(2):147–66.

[CR32] Gill R. Empowerment/sexism: figuring female sexual agency in contemporary advertising. Feminism Psychol. 2008;18(1):35–60.

[CR34] Hayes AF. Introduction to mediation, moderation, and conditional process analysis: A regression-based approach. New York: The Guilford Press; 2013.

[CR35] Heiserman N, Simpson B. Higher inequality increases the gap in the perceived merit of the rich and poor. Social Psychol Q. 2017;80(6):019027251771191.

[CR36] Hill SE, Rodeheffer CD, Griskevicius V, Durante K, White AE. Boosting beauty in an economic decline: mating, spending, and the lipstick effect. J Personal Soc Psychol. 2012;103(2):275–91. 10.1037/a0028657 22642483

[CR37] Hofstede G. Culture’s consequences: comparing values, behaviors, institutions and organizations across nations. 2nd ed. Thousand Oaks, CA: Sage; 2001.

[CR38] Hopkins-Doyle A, Sutton RM, Douglas KM, Calogero RM. Flattering to deceive: why people misunderstand benevolent sexism. J Personal Soc Psychol. 2019;116(2):167–92. 10.1037/pspa0000135 30359066

[CR39] Jackson T, Chen H. Sociocultural predictors of physical appearance concerns among adolescent girls and young women from China. Sex Roles. 2008;58:402–11.

[CR40] Jeffreys S. Beauty and misogyny: harmful cultural practices in the West. London: Routledge; 2005.

[CR41] Kellie DJ, Blake KR, Brooks RC. (2019). What drives female objectification? An investigation of appearance-based interpersonal perceptions and the objectification of women. PLoS ONE. 14(8):e0221388. 10.1371/journal.pone.0221388 10.1371/journal.pone.0221388 PMC6707629 31442260

[CR42] Kim JY, Fitzsimons GM, Kay AC. Lean in messages increase attributions of women’s responsibility for gender inequality. J Personal Soc Psychol. 2018;115(6):974–1001. 10.1037/pspa0000129 30550322

[CR43] Kowal M, Sorokowski P, Pisanski K, Valentova JV, Varella MA, Frederick DA, Mišetić K. Predictors of enhancing human physical attractiveness: data from 93 countries. Evol Hum Behav. 2022;43(6):455–74.

[CR44] Kozee HB, Tylka TL. A test of objectification theory with lesbian women. Psychol Women Q. 2007;30:348–57.

[CR45] Labunskaya V. Relationship between satisfaction and concern with own appearance and subjective Estimation of economic status. Behav Sci. 2019;10(1):9. 31861645 10.3390/bs10010009 PMC7016700

[CR46] McKinley NM, Hyde JS. The objectified body consciousness scale: development and validation. Psychol Women Q. 1996;20(2):181–215.

[CR47] Netchaeva E, Rees M. Strategically stunning: the professional motivations behind the lipstick effect. Psychol Sci. 2016;27(8):1157–68. 27356962 10.1177/0956797616654677

[CR48] Riley S, Evans A, Elliott S, Rice C, Marecek J. A critical review of postfeminist sensibility. Soc Pers Psychol Compass. 2017;11(12):e12367.

[CR49] Roberts T-A, Calogero RM, Gervais S. Objectification theory: continuing contributions to feminist psychology. In: Travis C, White J, editors. APA handbook of the psychology of women: history, theory, and battlegrounds. Volume 1. Washington, DC: American Psychological Association; 2018. pp. 249–72.

[CR50] Rojo-Ramos J, Polo-Campos I, García-Gordillo MÁ, Adsuar JC, Galán-Arroyo C, Gómez-Paniagua S. The importance of gender in body mass index, age, and body self-perception of university students in Spain. Sustainability. 2023;15(6):4848.

[CR51] Sánchez-Rodríguez Á, Willis GB, Jetten J, Rodríguez‐Bailón R. Economic inequality enhances inferences that the normative climate is individualistic and competitive. Eur J Social Psychol. 2019;49(6):1114–27.

[CR52] Scholz JK, Sicinski K. Facial attractiveness and lifetime earnings: evidence from a cohort study. Rev Econ Stat. 2015;97(1):14–28. 30505018 10.1162/REST_a_00435 PMC6261420

[CR53] Thompson ER. Development and validation of an internationally reliable short-form of the positive and negative affect schedule (PANAS). J Cross-Cult Psychol. 2007;38(2):227–42.

[CR54] Vaes J, Cristoforetti G, Ruzzante D, Cogoni C, Mazza V. Assessing neural responses towards objectified human targets and objects to identify processes of sexual objectification that go beyond the metaphor. Sci Rep. 2019;9(1):6699. 31040314 10.1038/s41598-019-42928-x PMC6491438

[CR55] Wang X, Chen H, Chen Z. Women’s self-objectification under competition when they believe sex is power. Arch Sex Behav. 2022;51(6):2837–54. 35861947 10.1007/s10508-022-02335-2

[CR56] Wen H. Buying beauty: cosmetic surgery in China. Hong Kong: Hong Kong University; 2013.

[CR57] Wolf N. The beauty myth: how images of beauty are used against women. New York: W. Morrow; 1990.

[CR58] World Economic Forum. (2024). *The global gender gap report 2024.* Geneva: World Economic Forum. Retrieved from https://www.weforum.org/reports/global-gender-gap-report-2024 (Accessed March 2025).

[CR59] Zion Market Research. (2020). Plastic surgery market report. Retrieved from https://www.zionmarketresearch.com/report/plastic-surgery-market (Accessed March 2025).

