# Supplementary material for: Empowered yet dehumanized: perceptions of women’s attractiveness in the context of gender earnings inequality
Source: BMC Psychol. 2025 Aug 19;13:946. doi: 10.1186/s40359-025-03306-7 (PMC12366408; doi:10.1186/s40359-025-03306-7)
Supplement: Supplementary file 1 — Supplementary Material 1 [file 40359_2025_3306_MOESM1_ESM.docx]

**Supplemental Online Materials for**

**Empowered yet dehumanized:**

**Attractiveness serves women in the context of gender earnings inequality**

**Study 1**

Table S1 Descriptive Statistics and Zero-Order Correlations of Measured Variables

| Variable | 1 | 2 | 3 | 4 | 5 | 6 | 7 | 8 | 9 | 10 | 11 |
| --- | --- | --- | --- | --- | --- | --- | --- | --- | --- | --- | --- |
| 1. Gender earnings inequality |  |  |  |  |  |  |  |  |  |  |  |
| 2. Perceived benefit of attractiveness in dating | .10 |  |  |  |  |  |  |  |  |  |  |
| 3. Perceived benefit of attractiveness in interview | .03 | .52^***^ |  |  |  |  |  |  |  |  |  |
| 4. Beauty is power belief | .14^*^ | .48^***^ | .59^***^ |  |  |  |  |  |  |  |  |
| 5. Age | .01 | .11 | .16^*^ | .15^*^ |  |  |  |  |  |  |  |
| 6. BMI | .05 | .09 | .17^*^ | .13 | .03 |  |  |  |  |  |  |
| 7. Education | -.06 | -.08 | .08 | -.02 | .18^*^ | -.06 |  |  |  |  |  |
| 8. Income | -.12 | .14 | .01 | .02 | .10 | -.19^**^ | .24^**^ |  |  |  |  |
| 9. Subjective social class | -.04 | .03 | -.004 | -.02 | -.08 | -.09 | .11 | .38^***^ |  |  |  |
| 10. Identity | -.31^***^ | .12 | .23^**^ | .25^***^ | .08 | -.03 | .08 | .12 | -.02 |  |  |
| 11. Negative affect | .24^**^ | -0.1 | -.14^*^ | -.11 | .06 | .11 | -.05 | -.18^*^ | -.09 | -.40^***^ |  |
| *M* | - | 5.07 | 5.05 | 3.53 | 23.25 | 21.66 | 2.87 | 4.38 | 4.71 | 4.31 | 2.61 |
| *SD* | - | 1.1 | 1.28 | 1.24 | 3.85 | 6.08 | .65 | 1.66 | 1.38 | 2.02 | 0.49 |

*Note.* ^*^*p* < .05, ^**^*p* < .01, ^***^*p* < .001. For education, 1 = *Middle school and below*, 2 = *High school,* 3*= Bachelor’s degree,* 4 *= Master’s degree,* 5 *= Doctorate and above.* For income, 1 = *Less than 10,000 ¥*, 2 = *10,000-30,000 ¥*, 3 = *30,000-50,000 ¥*, 4 = *50,000-100,000 ¥*, 5 = *100,000-150,000 ¥*, 6 = *150,000-300,000 ¥*, 7 = *300,000-500,000 ¥*, 8 = *500,000-1,000,000 ¥*, 9 = *More than 1,000,000 ¥.*

#### Perceived benefit of attractiveness

Using ANOVAs, we found that in the dating context, participants in the gender earnings inequality condition (*M* = 5.17, *SD* = 1.11) did not differ with those in the control condition (*M* = 4.96, *SD* = 1.07), *F*(1, 198) = 1.82, *p* = .18, ηp^2^ = .01. Additionally, in the job interview context, participants in the gender earnings inequality condition (*M* = 4.09, *SD* = 1.31) did not differ with those in the control condition (*M* = 4.01, *SD* = 1.26), *F*(1, 198) = .21, *p* = .65, ηp^2^ = .001.

Furthermore, participants in the gender earnings inequality condition (*M* = 3.70, *SD* = 1.24) reported a higher belief in the idea that beauty is power than those in the control condition (*M* = 3.34, *SD* = 1.23), *F*(1, 198) = 4.15, *p* = .04, ηp^2^ = .021.

#### Exploratory mediation effect of beauty is power belief

We conducted bootstrapping mediation analyses with 5000 iterations (Model 4; Hayes, 2013). Specifically, we modeled the condition (gender earnings inequality versus control) as the independent variable, belief in the idea that “beauty is power” as the mediator, and the perceived benefit of attractiveness in the interview and dating as dependent variables.

The results showed that the indirect effect of “beauty is power” belief on the relationship between the gender earnings inequality and the perceived benefit of attractiveness were significant in both the interview (*b* = .22, *SE* = .11, 95% CI [.01, .43]) and the dating context (*b* = .15, *SE* = .07, 95% CI [.01, .29]). Specifically, when predicting the perceived benefit of attractiveness in interviews, gender earnings inequality significantly predicted the “beauty is power” belief (*b* = .36, *SE* = .17, *p* = .04, 95% CI [.01, .70]). Additionally, controlling for gender earnings inequality, the “beauty is power” belief significantly predicted the perceived benefit of attractiveness in interviews (*b* = .62, *SE* = .06, *p* < .001, 95% CI [.50, .74]). However, the direct effect of gender earnings inequality on the perceived benefit of attractiveness in interviews did not reach significant (*b* = -.14, *SE* = .15, *p* = .35, 95% CI [-.43, .15]; see Figure S1a). Similarly, when predicting the perceived benefit of attractiveness in dating, gender earnings inequality significantly predicted the “beauty is power” belief (*b* = .36, *SE* = .17, *p* = .04, 95% CI [.01, .70]). Additionally, controlling for gender earnings inequality, the “beauty is power” belief significantly predicted the perceived benefit of attractiveness in dating (*b* = .42, *SE* = .06, *p* < .001, 95% CI [.31, .53]). However, the direct effect of gender earnings inequality on the perceived benefit of attractiveness in dating did not reach significant (*b* = .06, *SE* = .14, *p* = .66, 95% CI [3.15, 3.98]; see Figure S1b).


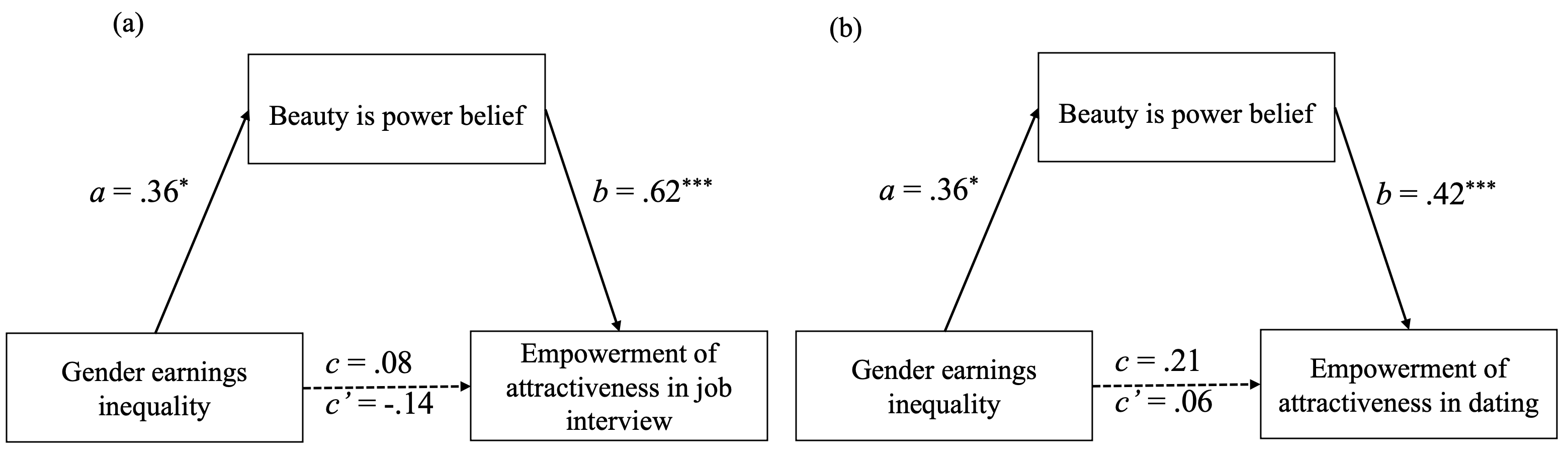


Figure S1. Beauty is power belief mediate the effect of gender earnings inequality on perceived benefit of attractiveness in job interview (a) and dating (b) in Study 1. *Note.* Unstandardized coefficients are displayed. The c path shows the total effect and the c′ path shows the direct effect. ^*^*p* < .05, ^***^*p* < .001.

**Study 2**

Table S2 Descriptive Statistics and Zero-Order Correlations of Measured Variables

| Variable | 1 | 2 | 3 | 4 | 5 | 6 | 7 | 8 | 9 | 10 | 11 |
| --- | --- | --- | --- | --- | --- | --- | --- | --- | --- | --- | --- |
| 1. Appearance concern |  |  |  |  |  |  |  |  |  |  |  |
| 2. Humanness | -.30^***^ |  |  |  |  |  |  |  |  |  |  |
| 3. Empowerment | -.17^*^ | .68^***^ |  |  |  |  |  |  |  |  |  |
| 4. Likelihood of objectification | .59^***^ | -.22^**^ | -.16^*^ |  |  |  |  |  |  |  |  |
| 5. Intention of making friends | -.31^***^ | .64^***^ | .52^***^ | -.13^†^ |  |  |  |  |  |  |  |
| 6. Age | .18^**^ | -.01 | -.09 | .13 | -.08 |  |  |  |  |  |  |
| 7. Gender | -.02 | .04 | -.02 | .04 | .05 | .09 |  |  |  |  |  |
| 8. BMII | -.04 | -.05 | -.09 | -.03 | .01 | .10 |  |  |  |  |  |
| 9. Education | -.10 | -.09 | -.03 | -.09 | <.001 | .09 | -.18^**^ |  |  |  |  |
| 10. Income | .03 | .12 | .07 | .01 | -.02 | .26^***^ | .09 | -.05 |  |  |  |
| 11. Subjective social class | .01 | .03 | .02 | .07 | -.02 | .08 | -.04 | -.01 | .18^**^ | .40^***^ |  |
| *M* | - | 4.35 | 3.80 | 4.53 | 4.13 | 27.17 | - | 22.81 | 2.96 | 4.72 | 4.74 |
| *SD* | - | 1.29 | 1.42 | 1.52 | 1.52 | 6.04 | - | 5.41 | .72 | 1.68 | 1.49 |

*Note.* ^*^*p* < .05, ^**^*p* < .01, ^***^*p* < .001. ^†^*p* = .051. For education, 1 = *Middle school and below*, 2 = *High school,* 3*= Bachelor’s degree,* 4 *= Master’s degree,* 5 *= Doctorate and above.* For income, 1 = *Less than 10,000 ¥*, 2 = *10,000-30,000 ¥*, 3 = *30,000-50,000 ¥*, 4 = *50,000-100,000 ¥*, 5 = *100,000-150,000 ¥*, 6 = *150,000-300,000 ¥*, 7 = *300,000-500,000 ¥*, 8 = *500,000-1,000,000 ¥*, 9 = *More than 1,000,000 ¥.*

Table S3 Summary of the results of Study 2

|  | Humanness | Empowerment | Likelihood of objectification | Intention of making friends |
| --- | --- | --- | --- | --- |
| Participant gender | *F*(1, 210) = .24  *p* = .63  η*_p_*^2^ = .001 | *F*(1, 210) = .16  *p* = .69  η*_p_*^2^ = .001 | *F*(1, 210) = .72  *p* = .40  η*_p_*^2^ = .003 | *F*(1, 210) = .54  *p* = .46  η*_p_*^2^ = .003 |
| Appearance concern | *F*(1, 210) = 20.95  *p<*.001^***^  η*_p_*^2^ = .103 | *F*(1, 210) = 6.45  *p* = .017^**^  η*_p_*^2^ = .027 | *F*(1, 215)=114.28  *p* < .001^***^  η*_p_*^2^ = .332 | *F*(1, 210) = 22.25  *p* < .001^***^  η*_p_*^2^ = .089 |
| Interaction effect | *F*(1, 210) = .60  *p* = .44  η*_p_*^2^ = .003 | *F*(1, 210) = .08  *p* = .78  η*_p_*^2^ < .001 | *F*(1, 210) = .61  *p* = .44  η*_p_*^2^ = .003 | *F*(1, 210) = .11  *p* = .74  η*_p_*^2^ = .001 |

*Note.* ^**^*p* < .01, ^***^*p* < .001.

#### Participants’ perceptions of the target’s concern with appearance

To examine the perceived effect of attractiveness in the gender earnings inequality, ACOVAs were conducted, with appearance concern (control condition = 0, appearance concern condition = 1) and the participant’s gender (man = 0, woman = 1) as the independent variables; humanness, empowerment, likelihood of objectification, and intention of making friends as the dependent variables.

It was demonstrated that neither the main effect of the participant’s gender nor the interaction effect reached significance when predicting humanness, empowerment, the likelihood of objectification, or the intention of making friends (all *p*s > .05), while the main effect of attractiveness concern was significant. Specifically, participants in the appearance concern condition rating the target as less human (*F*(1, 215) = 20.95, *p* < .001, η*_p_*^2^ = .089; *M_appearance concern condition_* = 4.00, *SD* = 1.23; *M_control condition_* = 4.77, *SD* = 1.24), less empowered (*F*(1, 215) *=* 6.45, *p* =.01, η*_p_*^2^ *=*.029; *M_appearance concern condition_* = 3.58, *SD* = 1.22; *M_control condition_* = 4.06, *SD* = 1.60), and more likely to encounter objectification in daily life (*F*(1, 215) *=* 114.28, *p* < .001, η*_p_*^2^ *=* .347; *M_appearance concern condition_* = 5.35, *SD* = .99; *M_control condition_* = 3.56, *SD* = 1.47), as well as less intention to make friends with the target (*F*(1, 215) *=* 22.25, *p* < .001, η*_p_*^2^ *=* .094; *M_appearance concern condition_* = 3.71, *SD* = 1.41; *M_control condition_* = 4.64, *SD* = 1.51).

#### Further analysis of Humanness

Further exploratory analyses conducted ANOVA with agency, competence, warmth, and morality as dependent variables (Table S4).

Table S4 Summary of the results of Study 2

|  | Agency | Competence | Warmth | Morality |
| --- | --- | --- | --- | --- |
| Participant gender | *F*(1, 215) = .02  *p* = .88  η*_p_*^2^ < .001 | *F*(1, 215) = .22  *p* = .64  η*_p_*^2^ = .001 | *F*(1, 215) = 0.02  *p* = .88  η*_p_*^2^ < 0.001 | *F*(1, 215) = .50  *p* = .48  η*_p_*^2^ = .002 |
| Appearance concern | *F*(1, 215) = 25.86  *p<*.001^***^  η*_p_*^2^ = .11 | *F*(1, 215) = 37.38  *p* < 0.001  η*_p_*^2^ = .15 | *F*(1, 215)= 25.86  *p* < .001^***^  η*_p_*^2^ = .11 | *F*(1, 215) = 44.68  *p* < .001^***^  η*_p_*^2^ = .17 |
| Interaction effect | *F*(1, 215) = .46  *p* = .50  η*_p_*^2^ = .002 | *F*(1, 215) < 0.001  *p* = 1.00  η*_p_*^2^ < .001 | *F*(1, 215) = .46  *p* = .05  η*_p_*^2^ = .002 | *F*(1, 215) < 0.001  *p* = .99  η*_p_*^2^ < 0.001 |

***Agency***

The main effect of participant gender was not significant, *F*(1, 215) = 0.02, *p* = 0.88, η_p_² < 0.001. However, the main effect of appearance concern was significant, *F*(1, 215) = 25.86, *p* < 0.001, η_p_² = 0.11. Participants in the appearance concern condition (*M* = 5.07, *SD* = 0.16) perceived the target as having a higher level of agency compared to those in the control condition (*M* = 3.98, *SD* = 0.15). The interaction between participant gender and appearance concern was not significant, *F*(1, 215) = 0.46, *p* = 0.50, η_p_² = 0.002.

***Competence***

The main effect of participant gender was not significant, *F*(1, 215) = 0.22, *p* = 0.64, η_p_² = 0.001. However, the main effect of appearance concern was significant, *F*(1, 215) = 37.38, *p* < 0.001, η_p_² = 0.15. Participants in the appearance concern condition (*M* = 3.87, *SD* = 0.13) perceived the target as having a lower level of competence compared to those in the control condition (*M* = 5.05, *SD* = 0.14). The interaction between participant gender and appearance concern was not significant, *F*(1, 215) < 0.001, *p* = 1.00, η_p_² < 0.001.

***Warmth***

The main effect of participant gender was not significant, F(1, 215) = 0.02, p = 0.88, η_p_² < 0.001. However, the main effect of appearance concern was significant, F(1, 215) = 25.86, p < 0.001, η_p_² = 0.11. Participants in the appearance concern condition (M = 5.07, SD = 0.16) perceived the target as having a higher level of agency compared to those in the control condition (M = 3.98, SD = 0.15). The interaction between participant gender and appearance concern was not significant, F(1, 215) = 0.46, p = 0.50, η_p_² = 0.002.

***Morality.*** The main effect of gender pay inequality was not significant, F(1, 215) = 0.50, p = 0.48, η_p_² = 0.002. However, the main effect of appearance concern was significant, F(1, 215) = 44.68, p < 0.001, η_p_² = 0.17. Participants in the appearance concern condition (M = 3.78, SD = 0.12) perceived the target as having a lower level of morality compared to those in the control condition (M = 4.99, SD = 0.13). The interaction between participant gender and appearance concern was not significant, F(1, 215) < 0.001, p = 0.99, η_p_² < 0.001.

**Supplementary Study: Outside observers’ perception of women’s appearance concern in the gender earnings equality context**

This study aimed to explore whether third-party observers perceive appearance concern as a form of empowerment for women **in the gender earnings equality context**.

#### Methods

#### *Participants*

An *a priori* sample size analysis for a two-factor between-subjects ANOVA showed that at least 219 participants were needed to detect a small-to-medium effect size (η*p*2 = .035), with a statistical power of 80% and an alpha level of .05 (Faul et al., 2007). A total of 234 heterosexual Chinese participants (*M_age_* = 26.85 years, *SD* = 5.73; men, 51.3%) were recruited from Weidiaocha. All participants gave informed consent prior to their participation and received a small amount of compensation at the end of the study.

#### *Procedure*

The participants were informed that the study aimed to examine the effect of impression management. To manipulate the gender earnings inequality, participants were shown three pie charts depicting the wealth distributions between men and women in three anonymous countries (Countries *M*, *L*, and *K*; Heiserman & Simpson, 2017). To avoid making assumptions about other features of the three countries, participants were informed that the countries have similar GDPs per capita, political systems, religious beliefs and levels of economic inequality. Participants were then told that they would answer questions about Country *M*, which, depending on the condition, was described as having either a high or low gender earnings inequality relative to the other two countries. In country M/K (with high gender earnings inequality), men account for 85% of the average salary, while women account for 15%. In country L (with a moderate gender earnings inequality), men account for 72% of the average salary, while women account for 28%. In country K/M (control condition, with low gender earnings inequality), men receive 52% of the total pay, while women receive 48%. **However, in the present study, all participants were assigned to the gender earnings equality condition.**

To check participants’ comprehension, they completed two manipulation check items: “Which country has the most unequal distribution of pay by gender?” and “Which country has the most equal distribution of pay by gender?” (1 *= M,* 2 *= L,* 3 *= K*). Only participants who answered these questions correctly were allowed to proceed with the study.

Next, participants were randomly assigned to either the appearance concern condition (*n* = 105, 49.5% female) or the control condition (*n* = 129, 48.1% female; adapted from De Wilde et al., 2021, Study 2). The participants were shown a *Body Attitude Scale* consisting of 10 items (adopted from Calogero et al., 2017; McKinley & Hyde, 1996; e.g., “I often worry about whether the clothes I am wearing make me look good.” 1 *= completely disagree,* 7 *= completely agree*), completed by a woman named *Ms. A* who lives in country *M*. In the appearance concern condition, participants were shown that *Ms. A* was more concerned about her appearance than her competence (i.e., with 5 items rated as *agree*, and another 5 items as *completely agree*). In the control condition, participants were shown that *Ms. A* was more concerned about her competence than her appearance, i.e., with less self-objectification (i.e., with 5 items rated as *disagree* and another 5 items rated as *completely disagree*). The manipulation check was assessed using a single item: “To what extent do you think *Ms. A* is concerned about their appearance?” (1 = *not at all*, 7 = *extremely*). To enhance the manipulation effect, participants were instructed to write a short essay about their thoughts on *Ms. A*.

Next, participants completed the measures of the dependent variables, including the target’s humanness, perceived empowerment of the target, likelihood of sexual objectification in daily life, and intentions to befriend the target. Finally, participants reported their demographic information and affect, identical to Study 1, before being thanked and debriefed. However, we did not collect participants’ identification with the country in Study 2, as the manipulation of gender earnings inequality was not suitable for measuring identification.

#### *Measures*

*Humanness.* Sixteen attributes were used to rate the humanness of the target across four aspects: agency (assertive, independent, ambitious, and determined), warmth (warm, friendly, sociable, and likeable), competence (competent, clever, efficient, and capable), and morality (honest, sincere, trustworthy, and righteous) (De Wilde et al., 2021). Participants were asked, “To what extent do you perceive the woman as having the following attributes?” Each aspect was measured with 4 items (1 *= Completely disagree,* 7 *= Completely agree*). The average score was calculated, with a higher score indicating greater perceived humanness of the target (*α* = .94).

*Perceived empowerment of the target.* Participant’s perceived empowerment of the target was measured using seven items adapted from Kim et al. (2018). An example item was “In country *M*, is *Ms. A* in control of her life?” (1 *= Strongly disagree*, 7 *= Strongly agree*). The average score was used as an indicator of perceived empowerment, with a higher score reflecting a higher degree of perceived empowerment (*α* = .90).

*Likelihood of sexual objectification in daily life.* The Interpersonal Sexual Objectification Scale (Kozee & Tylka, 2007) was used to measure the perceived likelihood of sexual objectification of the target in daily life. The participants rated 15 items assessing the frequency of sexual objectification the target encountered in daily life while living in country *M* using a 7-point scale (e.g., “being touched or caressed against her will”; 1 *= Never*, 7 *= All the time*). The average score was calculated as an indicator of the likelihood of objectification, with a higher score indicating a greater likelihood of being objectified (*α* = .97).

*Intentions to befriend the target.* Four items were used to measure participants’ intentions to befriend the target (e.g., “To what extent do you want to make friends with *Ms. A*?” 1 *= Not at all*, 7 *= Very much*). The average score was calculated, with a higher score indicating a stronger intention to befriend the target (*α* = .91).

*Control variables.* The PANAS-SF (*α_PA_* = .66, *α_NA_* = .73) and demographic information were collected as in Study 1.

### Results and discussion

#### *Manipulation check*

The independent-samples *t* test showed that the participants in the appearance concern condition (*M* = 6.71, *SD* = .48) reported significantly higher self-objectification than those in the control condition (*M* = 2.10, *SD* = 1.33), *t*(232) = 33.74, *p* < .001, 95% CI [4.43, 4.95], Cohen’s *d* = -4.43, indicating a successful manipulation.

#### *Participants’ perceptions of the target’s concern with appearance*

To examine the perceived effect of attractiveness in the context of gender earnings inequality, a between-participants MANOVA was conducted to assess the extent to which the appearance concern (control condition = 0, appearance concern condition = 1) and the participant gender (male = 0, female = 1) influenced the dependent variables (humanness, empowerment, likelihood of objectification, and intention to form friendships). Demographic variables and affect were controlled as covariates. The multivariate test revealed a significant main effect of appearance concern, *λ* = .49, *F*(4, 222) = 56.81, *p* < .001, η*_p_*^2^ = .506.

Table S5 Summary of the results of supplementary Study

|  | Humanness | Empowerment | Likelihood of objectification | Intention of making friends |
| --- | --- | --- | --- | --- |
| Participant gender | *F*(1, 225) = 6.50  *p* = .01  η*_p_*^2^ = .028 | *F*(1, 225) = 5.24  *p* = .02  η*_p_*^2^ = .02 | *F*(1, 225) = 30.91  *p* < .001  η*_p_*^2^ = .121 | *F*(1, 225) = 4.29  *p* = .04  η*_p_*^2^ = .019 |
| Appearance concern | *F*(1, 225) = 64.53  *p<*.001^***^  η*_p_*^2^ = .223 | *F*(1, 225) = 57.26  *p<*.001^***^  η*_p_*^2^ = .203 | *F*(1, 225)=174.29  *p* < .001^***^  η*_p_*^2^ = .437 | *F*(1, 225) = 30.48  *p* < .001^***^  η*_p_*^2^ = .119 |
| Interaction effect | *F*(1, 225) = .72  *p* = .40  η*_p_*^2^ = .003 | *F*(1, 225) = .28  *p* = .60  η*_p_*^2^ = .001 | *F*(1, 225) = .17  *p* = .68  η*_p_*^2^ = .001 | *F*(1, 225) = 2.29  *p* = .13  η*_p_*^2^ = .010 |

*Note.* ^**^*p* < .01, ^***^*p* < .001.

***Humanness.*** The results of the analysis of variance indicated a significant main effect of participant gender, *F*(1, 225) = 6.50, *p* = .01, η*_p_*^2^ = .028. Female participants (***M*** = 5.03, ***SD*** = 0.10) rated the target in the gender pay equality condition as having higher humanization traits compared to male participants (***M*** = 4.68, ***SD*** = 0.10). There was also a significant main effect of appearance concern, *F*(1, 225) = 64.53, *p<*.001, η*_p_*^2^ = .223. Participants in the appearance concern condition (***M*** = 4.30, ***SD*** = 0.10) rated the target as having lower levels of humanization than those in the control condition (***M*** = 5.41, ***SD*** = 0.09). However, the interaction effect between participant gender and appearance concern was not significant, *F*(1, 225) = .72, *p* = .40, η*_p_*^2^ = .003.

***Empowerment.*** The results of the analysis of variance indicated a significant main effect of participant gender, *F*(1, 225) = 5.24, *p* = .02, η*_p_*^2^ = .02. Female participants (***M*** = 5.14, ***SD*** = 0.11) rated the target in the gender pay equality condition as having higher humanization traits compared to male participants (***M*** = 4.80, ***SD*** = 0.11). There was also a significant main effect of appearance concern, *F*(1, 225) = 57.26, *p<*.001, η*_p_*^2^ = .203. Participants in the appearance concern condition (***M*** = 4.40, ***SD*** = 0.11) rated the target as having lower levels of empowerment than those in the control condition (***M*** = 5.54, ***SD*** = 0.10). However, the interaction effect between participant gender and appearance concern was not significant, *F*(1, 225) = .28, *p* = .60, η*_p_*^2^ = .001.

***Likelihood of objectification.*** The results of the analysis of variance indicated a significant main effect of participant gender, *F*(1, 225) = 30.91, *p* < .001, η*_p_*^2^ = .121. Female participants (***M*** = 3.08, ***SD*** = 0.10) rated the target in the gender pay equality condition as less likely to encounter objectification compared to male participants (***M*** = 3.87, ***SD*** = 0.10). There was also a significant main effect of appearance concern, *F*(1, 225) = 174.29, *p* < .001, η*_p_*^2^ = .437. Participants in the appearance concern condition (***M*** = 4.41, ***SD*** = 0.11) rated the target as more likely to encounter objectification than those in the control condition (***M*** = 2.54, ***SD*** = 0.10). However, the interaction effect between participant gender and appearance concern was not significant, *F*(1, 225) = .17, *p* = .68, η*_p_*^2^ = .001.

***Intention of making friends.*** The results of the analysis of variance indicated a significant main effect of participant gender, *F*(1, 225) = 4.29, *p* = .04, η*_p_*^2^ = .019. Female participants (***M*** = 4.67, ***SD*** = 0.13) rated the target in the gender pay equality condition as more likely to form friendships with the target compared to male participants (***M*** = 4.31, ***SD*** = 0.13). There was also a significant main effect of appearance concern, *F*(1, 225) = 30.48*, p* < .001, η*_p_*^2^ = .119. Participants in the appearance concern condition (***M*** = 4.00, ***SD*** = 0.13) rated the target as more likely to form friendships with the target than those in the control condition (***M*** = 3.98, ***SD*** = 0.12). However, the interaction effect between participant gender and appearance concern was not significant, *F*(1, 225) = 2.29*, p* = .13*,* η*_p_*^2^ = .010.
